# S07 Public green spaces as sustainable environments for inclusive HEPA

**DOI:** 10.1093/eurpub/ckae114.226

**Published:** 2024-09-26

**Authors:** Karim Abu-Omar, Antonina Tcymbal

**Affiliations:** Faculty of Sport and Health Sciences and Gerontology Research Center, University of Jyväskylä, Jyväskylä, Finland; Faculty of Sport and Health Sciences and Gerontology Research Center, University of Jyväskylä, Jyväskylä, Finland

## Abstract

As urban populations around the world continue to grow, the push to transform our cities into greener, more sustainable, and healthier places is gaining momentum. In this context, it is widely recognized that different characteristics of public green spaces influence who accesses them, and how active people are in them. The challenges of equality, volume, and quality of HEPA should thus be addressed by a comprehensive approach that is taking into consideration the characteristics of green space.

The symposium will contribute to an increased understanding of the role of physical environment and characteristics of green spaces to support inclusiveness and sustainability of HEPA. It foresees four presentations addressing the development of the urban environment. Two of which are research-oriented and present which spatial features of green spaces are particularly relevant for promoting PA in the vulnerable groups of older adults and teenagers. Two of which are practice-oriented and address two complementary sides of ensuring environmental quality and sustainability in relation to HEPA as a determinant of quality of life, health, and well-being.

